# Electrospun core–shell nanofibres embedding drug nanocrystals for long-acting mucosal delivery

**DOI:** 10.1016/j.mtbio.2026.103653

**Published:** 2026-09-08

**Authors:** Gaia Zucca, Barbara Vigani, Caterina Valentino, Lucia Lopez-Vidal, Martina Sangalli, Marco Ruggeri, Giuseppina Sandri, Silvia Rossi, Alejandro J. Paredes

**Affiliations:** a Department of Drug Sciences, University of Pavia, Viale Taramelli 12, Pavia, 27100, Italy; b School of Pharmacy, Queens University Belfast, Medical Biology Centre, 97 Lisburn Road, Belfast, BT9 7BL, United Kingdom

**Keywords:** Nanoparticles, Nanosuspensions, Media milling, Oral delivery, Solid drug nanoparticles

## Abstract

Oral mucositis (OM) is a common and painful complication of cancer therapies, compromising oral functions and quality of life. Traditional local treatments are limited by rapid clearance, poor local drug availability, and frequent reapplication, highlighting the need for mucoadhesive polymeric platforms enabling localized and sustained drug delivery. In this study, naringenin-nanocrystals (NAR-NCs) were produced via wet media milling to enhance NAR solubility and dissolution and subsequently encapsulated into core–shell electrospun nanofibres (NFs), with a hydrophilic poly(vinyl alcohol) (PVA)/Sangelose® (SG) core for NAR-NC stabilization and a polycaprolactone (PCL) shell for controlled release. A mucoadhesive coating composed of ethyl cellulose (EC) and SG was applied to the NAR-NC_NFs to prolong mucosal residence, protect against mechanical stress, and facilitate buccal administration. NAR-NCs exhibited a mean hydrodynamic diameter of ∼280 nm, narrow size distribution, high stability over 10 days, and a 3.6-fold increase in saturation solubility compared to raw NAR. Core–shell NFs displayed uniform, bead-free morphology with clear core–shell architecture. NAR-NCs encapsulated within NFs preserved their size after fibres dissolution, within the 200–500 nm range, suitable for buccal application and maintained tensile properties comparable to unloaded NFs. Release studies demonstrated that NAR-NCs dissolved rapidly (∼45 min), NAR-NCs_core NFs provided controlled release over 6 h, and the complete NAR-NCs_NFs system prolonged drug release up to 72 h. The system also demonstrated preliminary anti-inflammatory properties, as confirmed by IL-8 enzyme-linked immunosorbent assay (ELISA). The EC/SG_NFs prototypes maintained normal human dermal fibroblasts (NHDF) viability above 95%, supporting their suitability as a mucoadhesive, mechanically robust and biocompatible platform for oral mucositis treatment.

## Highlights

• Drug nanocrystals were successfully encapsulated into core–shell nanofibres.
• Nanocrystals preserved their size after electrospinning and fibre dissolution.
• A mucoadhesive coating increased mechanical stability.
• The platform sustained naringenin release for up to 72 h.
• The system showed excellent cytocompatibility and anti-inflammatory activity.

## Graphical abstract

**Figure:**
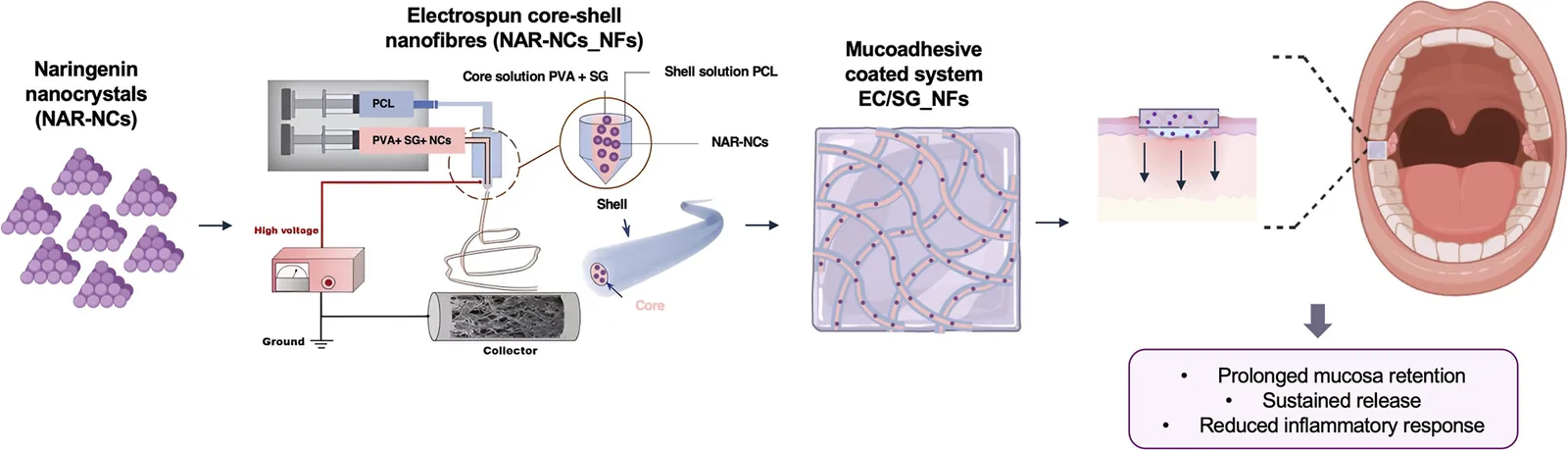

## 1 Introduction

Oral health is a fundamental component of overall wellbeing, enabling essential functions, such as eating, speaking, tasting, and smiling, while significantly influencing social and emotional quality of life [1,2]. Any discomfort or pathology in the oral cavity can therefore impact both physical and psychosocial aspects. Despite being largely preventable, oral diseases remain a major global health burden, affecting nearly 3.5 billion people worldwide and generating substantial healthcare costs. Common and often debilitating conditions include periodontal disease, oral cancers, candidiasis, xerostomia, recurrent aphthous stomatitis, lichen planus, and oral mucositis (OM) [1,3]. OM is a frequent and severe complication of treatments for head and neck cancers, particularly chemotherapy, immunotherapy, and radiotherapy [4]. Clinically, it is characterized by erythema, inflammation, and painful ulcerations that compromise chewing, swallowing, and speech, ultimately reducing patient quality of life. Moreover, mucosal barrier disruption increases susceptibility to opportunistic infections, while therapy-induced xerostomia can exacerbate symptoms and predispose patients to further oral complications [5,6].

Current therapies for OM primarily aim to alleviate symptoms through the administration of analgesics, local anaesthetics, antifungals, antivirals, or palliative measures such as saline rinses and protective gels [7]. In this scenario, traditional local dosage forms, such as gels, lozenges, and buccal tablets, are hindered by limited residence time in the oral cavity, which reduces the contact between the drug and the site of action. As a result, bioavailability remains low. Additional drawbacks, such as accidental swallowing, poor taste acceptance, and the need for frequent reapplication further limit therapeutic efficacy. These shortcomings collectively reduce patient adherence and compromise therapeutic effectiveness [8,9]. Given that the reduced residence time of traditional local dosage forms leads to poor bioavailability and, ultimately, suboptimal therapeutic outcomes, advanced therapeutic tools, such as nanostructured delivery systems, have gained increasing attention to improve the performance of certain drugs. Systems such as polymeric nanoparticles, nanofibres, liposomes, nanogels, dendrimers, and nanocrystals can improve drug solubility, protect it from enzymatic degradation, improve mucopenetration, and enable localized and sustained release [10,11]. Nanocrystals (NCs), in particular, have emerged as a promising strategy for the delivery of poorly water-soluble drugs [[12], [13], [14]]. NCs are submicron-sized pure drug dispersions (typically 100–1000 nm), produced by wet milling or high-pressure homogenization in the presence of stabilizers [13,14]. Their nanoscale size increases surface area, dissolution rate and saturation solubility of the drug particles, while also improving mucoadhesion, thereby promoting localized drug accumulation and reducing systemic exposure [13]. Despite these benefits, NCs administered directly into the oral cavity are susceptible to rapid clearance due to saliva and mechanical stress, which can compromise their therapeutic efficacy [15]. A promising strategy to overcome this drawback involves the incorporation of NCs into mucoadhesive polymeric carriers, such as electrospun nanofibres (NFs), which can be developed as patches for buccal application [15,16]. In the treatment of OM, after application, these fibrous mats adhere to the mucosal surface, protect the lesion, prolong residence time and ensure localized drug release [16]. Among these, core–shell NFs produced via coaxial electrospinning offer key advantages: the shell provides mechanical stability and controlled release, while the core enables the encapsulation of high drug loads or colloidal formulations [17]. Upon contact with saliva, hydration of the polymeric fibrous matrix could progressively enable drug release, while the enhanced saturation solubility and dissolution rate provided by NCs promote local drug availability at the mucosal surface [18].

Given these premises, the present work aimed to develop core–shell NFs loaded with naringenin nanocrystals (NAR-NCs), subsequently coated with a mucoadhesive film for the local treatment of OM. NAR, a natural flavonoid abundant in citrus fruits, is well known for its anti-inflammatory and antioxidant properties; however, its clinical application is hindered by poor aqueous solubility. To address these issues, NAR-NCs were produced to enhance NAR dissolution and bioavailability. The core-shell NFs were designed with a hydrophilic poly (vinyl alcohol) (PVA)/Sangelose® (SG) core for NAR-NCs stabilization and a polycaprolactone (PCL) shell for sustained release. A mucoadhesive coating composed of ethyl cellulose (EC) and SG was applied onto the NCs-in-NFs system to promote prolonged mucosal residence, protect against mechanical stress and accidental swallowing, and provide modular control over adhesion independently of the drug release governed by the core–shell architecture.

To the best of our knowledge, this is the first study to formulate NAR-NCs for the local treatment of OM and to successfully incorporate them into NFs via coaxial electrospinning. The use of a core–shell design is particularly novel, as it enables for the first time the encapsulation of drug NCs within the fibre core, while maintaining mechanical stability and controlled release through the shell. In addition, we report the first integration of NCs-in-NFs with a mucoadhesive film, representing an innovative therapeutic platform for buccal application that combines controlled release, protection against mechanical stress, and prolonged mucosal residence. Finally, this work reports for the first time the use of SG in combination with PVA in electrospinning, thereby opening new perspectives for its application in drug delivery.

## 2 Materials and methods

### 2.1 Materials

Naringenin (NAR; LKT Laboratories, Inc., UK) and Poloxamer P188 (BASF Pharma, UK) were used for NCs preparation. Poly(vinyl alcohol) (PVA, 85–124 kDa; Sigma-Aldrich, UK) and Sangelose® (SG) 60L (MW 400,000 Da; KISKO Deutschland GmbH, Germany) were employed for the core of the core–shell nanofibres, while polycaprolactone (PCL, 50 kDa; Ingevity, UK) was used for the shell. Simulated saliva fluid (SSF) was prepared using phosphate-buffered saline (PBS; Sigma-Aldrich, UK) and Tween 80 (Ases Chemical Works, UK). For the preparation of the mucoadhesive film, Ethocel™ Standard 100 Premium ethyl cellulose (EC; Colorcon Limited, UK), SG 60L and 90L (MW 600,000 Da; KISKO Deutschland GmbH, Germany), and polyethylene glycol (PEG 2000; Sigma-Aldrich, UK) were used. Ultrapure water was obtained from a water purification system, Elga Purelab DV 25, Veolia Water Systems (Ireland). All other chemicals used were of analytical grade.

### 2.2 Methods

#### 2.2.1 Naringenin-nanocrystals preparation

Following the method described by Lopez-Vidal et al., naringenin-nanocrystals (NAR-NCs) were prepared via a top-down approach, namely wet media milling, using a dual asymmetric centrifuge (DAC) (FlackTek™ 330-100 SE, Louisville, Colorado, USA) [19]. Briefly, 100 mg of NAR and 3 mL of yttria-stabilized zirconia (YTZP) milling beads (Chemco, UK) (diameter 0.10–0.15 mm) were placed in a 12 mL polypropylene container (density 0.905 g/cm^3^; SinergyMixers®) together with 7 mL of a 1% w/v P188 stabilizer solution. The suspension was processed at 3000 rpm in 3-min cycles, repeated six times (total milling time: 18 min), with all process parameters and equipment settings kept constant. After milling, the suspension was centrifuged using a Sigma benchtop micro-centrifuge (SciQuip Ltd, Shropshire, England) at 7379 x g for 10 min to remove the excess P188 and concentrate the NCs. The supernatant was discarded, and the pellet was subsequently resuspended in ultrapure water for further analysis.

#### 2.2.2 Naringenin-nanocrystals characterization

##### 2.2.2.1 Laser diffraction and dynamic light scattering

The particle size of the raw NAR powder prior to milling was determined by laser diffraction (LD) using a Malvern Mastersizer® 3000 (Malvern Panalytical Ltd., UK). For sample preparation, 10 mg of NAR powder was dispersed in 10 mL of a 1% w/v P188 solution and vortex-mixed for 5 min to obtain a uniform suspension. The dispersion was transferred into a 500 mL beaker containing ultrapure water. During LD analysis, it was stirred at 2000 rpm for 3 min, with 30 s of sonication applied between measurement cycles to ensure stable and reproducible dispersion. For NAR-NCs, particle size and polydispersity index (PDI) were determined before and after the centrifugation by dynamic light scattering (DLS) using a Nanobrook Omni analyser (Brookhaven Instruments Corp., USA). For analysis, 20 μL of the nanosuspension was diluted in 3 ml of ultrapure water, and measurements were conducted based on intensity-weighted size distribution.

##### 2.2.2.2 Stability

The stability of NAR-NCs was assessed by storing the samples at room temperature (25 °C) under light-protected conditions for 10 days. Particle size was determined by DLS (as reported in Section 2.2.1), before and after centrifugation, at the beginning and end of the storage period. Variations in size were used as indicators of NAR-NCs stability and to assess the impact of the centrifugation process on particle integrity.

##### 2.2.2.3 Scanning and transmission electron microscope

The morphology of raw NAR powder was examined by scanning electron microscopy (SEM) using a SNE-ALPHA microscope (SEC Co., Ltd., KR). The powder was mounted on double-sided carbon adhesive tape affixed to aluminium stubs and observed at an accelerating voltage of 5 kV with a magnification of 500×. For NAR-NCs visualization, transmission electron microscopy (TEM) was performed using a JEM-1400 Plus microscope (JEOL, J) operating at 100 kV and equipped with a JEOL Ruby 8 MP bottom-mounted CCD digital camera. A drop of the NAR-NCs nanosuspension was deposited onto a 300-mesh copper grid coated with a pure carbon film and allowed to dry at room temperature for 1 h prior to imaging.

##### 2.2.2.4 Differential scanning calorimetry and thermogravimetric analysis

The thermal behaviour of NAR-NCs, their individual components (raw NAR and P188), and the corresponding physical mixture (PM) was evaluated by differential scanning calorimetry (DSC) using a Q20 system (TA Instruments, USA). The PM was prepared by accurately weighing raw NAR and P188 in the same ratios NCs preparation and homogenizing them in DAC using the same operating parameters, but in the absence of milling beads, to avoid particle size reduction. Approximately 8 mg of each sample was placed in non-hermetically sealed aluminium pans and analysed at a heating rate of 5 °C/min over a temperature range of 25–300 °C under a dynamic nitrogen flow (50 mL/min). Prior to analysis, the instrument was calibrated with indium according to the manufacturer's protocol. Thermogravimetric analysis (TGA) was performed on a Q50 Thermogravimetric Analyzer (TA Instruments, UK). Samples were heated from 30 °C to 350 °C at 5 °C/min under nitrogen atmosphere, with flow rates of 50 mL/min for both balance and sample purge gases. Data acquisition and processing were carried out using Universal Analysis 200 software, version 4.4A (TA Instruments, UK).

##### 2.2.2.5 Infrared spectroscopy

Fourier-transform infrared (FT-IR) spectra of NAR-NCs and raw powders were acquired using a Spectrum Two FT-IR spectrometer (PerkinElmer, MA, USA) equipped with a MIRacle™ Single Reflection attenuated total reflectance (ATR) accessory and a MIRacle™ Confined Space Clamp (Pike Technologies, MA, USA). Spectra were collected over the range of 4000–600 cm^−1^ at a resolution of 4 cm^−1^, averaging 32 scans for each sample.

##### 2.2.2.6 Drug content

The drug content of NAR-NCs was determined by spectrophotometric analysis using a Microplate Reader (FLUOstar Omega, BMG LABTECH, DE). Briefly, 50 μL of NAR-NCs nanosuspension, obtained after centrifugation (10,000 rpm, 10 min) and resuspension in ultrapure water, was diluted 1:4 v/v with ethanol (Sigma-Aldrich, UK). The absorbance of the resulting solution was measured at 290 nm. To quantify the amount of NAR in the NAR-NCs, a calibration curve was generated (r^2^ = 0.997) using NAR solutions in ethanol at concentrations ranging from 0.00625 to 0.03 mg/mL. The drug content of NAR-NCs was expressed as the percentage of NAR recovered in the NCs pellet relative to the theoretical amount.

##### 2.2.2.7 Saturation solubility

The solubility of NAR-NCs and the corresponding PM was evaluated using a saturation solubility assay. An excess amount of each sample was added to 5 mL of ultrapure water and incubated for 48 h at 37 ± 0.1 °C in a temperature-controlled bath under continuous agitation at 20 rpm. Following incubation, suspensions were centrifuged and the supernatants filtered through 0.22 μm membrane filters (Millipore, USA). All experiments were performed in triplicate.

#### 2.2.3 Preparation of polymeric solutions for electrospinning

The preparation of the core–shell fibres involved the separate preparation of two stock solutions: one for the core (core solution) and one for the shell (shell solution). The core solution was composed of PVA and SG60. 20% w/v PVA solution was obtained by dissolving the polymer in ultrapure water at 85 °C in a water bath, under vigorous magnetic stirring for 2 h to ensure complete dissolution. SG60 solution was prepared according to the method described by Iohara et al. [20]. Briefly, SG60 was dissolved at a concentration of 2% w/v in ultrapure water, heated to 60 °C in a water bath, and stirred magnetically for 30 min. The solution was then left at room temperature for an additional 30 min to equilibrate. The PVA and SG60 solutions were subsequently mixed in a 1:1 v/v ratio to obtain core solution. The shell solution consisted of PCL dissolved at 30% w/v in 90% v/v acetic acid (Sigma-Aldrich, UK), with continuous magnetic stirring until a homogeneous solution was obtained.

#### 2.2.4 Core-shell nanofibres preparation and characterization

Core–shell nanofibres (NFs) were fabricated using a coaxial electrospinning setup (Fluidnatek® LE-100, Bioinicia S.L., ES) equipped with a coaxial spinneret connected to two independent syringe pumps. The shell solution was fed at a flow rate of 1.5 mL/h (shell solution), while the core solution was delivered at 0.8 mL/h (core solution). A positive voltage of 18.5 kV was applied to the spinneret, with the tip-to-collector distance set at 18 cm. Electrospinning was performed for 6 h under controlled environmental conditions using the integrated Environmental Control Unit (ECU), maintaining ambient temperature and a relative humidity of 40% throughout the process. Fibres were collected on a grounded collector until a uniform mat was obtained for further analyses.

The morphology of the core–shell NFs and their reference components (PVA/SG60 core and PCL shell) was examined using a scanning electron microscope (SEM; Phenom™ Pure Desktop, Thermo Scientific, USA). Fibre samples were mounted on steel stubs and sputter-coated under vacuum with a 10 nm graphite layer to enhance conductivity. SEM observations were performed at room temperature under high-vacuum conditions with an accelerating voltage of 5 kV. Images were captured at a magnification of 15 kX, and NF diameters were quantified using ImageJ™ 2.0 (U S. National Institute of Health, Bethesda, MD, USA). The internal structure of core–shell NFs was confirmed by TEM using a JEM-1400 Plus microscope (JEOL, J) operated at an accelerating voltage of 100 kV (n = 3). For TEM analysis, NFs were collected during the electrospinning process by directly depositing them onto copper grids coated with a carbon film.

#### 2.2.5 Naringenin-nanocrystals loading into core-shell nanofibres

To prepare the NAR-NCs loaded NFs (NAR-NCs_NFs), NAR-NCs dispersions from four independent batches were pooled, centrifuged, and the resulting pellets were resuspended in 0.5 mL of ultrapure water. This dispersion was then mixed with 10 mL of Core solution using a DAC 150 FVZ at 3000 rpm for 1 min to obtain a homogeneous dispersion. The coaxial electrospinning was then carried out using the same equipment setup and process parameters described in Section 2.2.4.

#### 2.2.6 Naringenin-nanocrystals-loaded core-shell nanofibres characterization

##### 2.2.6.1 Morphological evaluation

The morphology of NAR-NCs-loaded NFs (NAR-NCs_NF) was examined by TEM, as reported in Section 2.2.2.3. The analysis was performed only on nanofibres prepared from the core solution containing NAR-NCs (NAR-NCs_core; single-needle electrospinning configuration), since this sample could be completely solubilized in water. Specifically, ∼1 cm^2^ of nanofibre mat was dissolved in 1 mL of ultrapure water. Subsequently, 20 μL of the resulting suspension were diluted in 3 mL of ultrapure water prior to DLS analysis, following the procedure described in Section 2.2.2.1.

##### 2.2.6.2 Determination of drug content

For drug content determination, NAR-NCs_NFs were dissolved in a 1:1 mixture of ultrapure water: 90% v/v acetic acid, and the resulting solution was diluted 1:4 v/v with ethanol before spectrophotometric quantification, as reported in Section 2.2.2.6. A separate calibration curve was prepared using NAR standard solutions in the same solvent system (0.00625–0.03 mg/mL; r^2^ = 0.995) to account for the different sample matrix.

##### 2.2.6.3 Mechanical properties evaluation

The mechanical properties of NFs and NAR-NCs_NFs were assessed using a TA.XT Plus Texture Analyzer (Stable Micro Systems, UK) equipped with a 50 kg load cell. Nanofibre mats were cut into strips measuring 1 × 3 cm^2^ and mounted between the instrument's tensile grips, with an initial grip separation set at 1 cm. The thickness of each specimen was measured immediately before testing using a Sicutool 3955G-50 thickness gauge. The cross-sectional area used to calculate the tensile strength was determined by multiplying the measured thickness by the specimen width of the strips. Tensile tests were performed by moving the upper grip upward at a constant rate of 5 mm/s until a maximum displacement of 200 mm was reached. Stress–strain curves were obtained from the recorded force–distance data. Stress was calculated by dividing the applied force by the initial cross-sectional area, while strain was calculated as the ratio between the extension and the initial gauge length. Young's modulus was determined by linear regression of the initial linear region of the stress–strain curves (2–8% strain), selected as the interval showing the best linearity for all samples. Since strain was expressed as %, slope values were converted to MPa by multiplying by 100. The parameters evaluated included maximum tensile strength, elongation at break and Young's modulus. Three replicates were analysed for each NF specimens.

##### 2.2.6.4 In vitro dissolution rate study

The *in vitro* dissolution profiles of raw NAR, NAR-NCs, NAR-NCs-loaded core NFs and NAR-NCs_NFs were analysed. Experiments were carried out under sink conditions, defined as maintaining the drug concentration below 10% of its saturation solubility, in accordance with standard guidelines [21]. Simulated saliva fluid (SSF), prepared by dissolving 2% w/v of Tween 80 in pH 6.4 PBS [15], was used as dissolution medium to provide sufficient solubilization capacity for the poorly water-soluble NAR. The saturation solubility of raw NAR and the different NAR-containing formulations was determined in this medium using the procedure described in Section 2.2.2.7. The dissolution-medium volume was subsequently selected based on these values to ensure sink conditions throughout the release study. At predetermined time intervals, 300 μL aliquots were withdrawn (not exceeding 3% of the total dissolution volume) and immediately replaced with an equal volume of fresh medium to maintain constant sink conditions. The collected samples were filtered through 0.22 μm membranes to remove undissolved particles prior to analysis, aiming to quantify the dissolved NAR fraction. Dissolution testing continued until complete drug release was achieved for NAR-NCs and NAR-NCs-loaded fibres (core and core-shell ones). In the case of raw NAR, the profiles were recorded up to its saturation solubility in SSF. The amount of NAR released was quantified spectrophotometrically at 290 nm, after 1:4 v/v dilution in ethanol. All experiments were performed in triplicate, and the cumulative release was expressed as the percentage of the initially loaded drug.

##### 2.2.6.5 In vitro studies on fibroblast cell line

Preliminary cytocompatibility studies of NFs were carried out *in vitro* using normal human dermal fibroblasts (NHDF, juvenile foreskin; PromoCell, I). Cells at passages 6–10 were cultured in complete medium (DMEM supplemented with 10% v/v fetal bovine serum and 1% v/v penicillin–streptomycin) at 37 °C in a humidified 5% CO_2_ atmosphere. Cytotoxicity was assessed according to ISO 10993-5:2009 recommendations, widely used for the biocompatibility evaluation of biomaterials and drug-delivery systems. Prior to testing, the fibres were sterilized by UV irradiation for 15 min. Extracts were prepared by incubating 10 mg of each scaffold in 1 mL of serum-free DMEM for 24 h at 37 °C. Cells grown in standard culture medium (CM) served as the 100% viability control. NHDF were seeded at a density of 35,000 cells/well in 96-well plates. After 24 h, the culture medium was removed and 200 μL of each integrated system extract was added to the wells. Following a 24-h exposure period, cell viability was evaluated using the Alamar Blue assay. Briefly, the extracts were removed and 200 μL of a 10% v/v Alamar Blue solution (Sigma-Aldrich, I) in DMEM was added to each well and incubated for 3 h. Fluorescence was then measured using a multimode microplate reader (FLUOstar Omega, BMG LabTech, DE) at 570 nm (reduced form) and 655 nm (oxidized form). Cell viability was expressed as a percentage relative to untreated controls. Each condition was tested in six replicates.

Anti-inflammatory properties were finally investigated. The levels of IL-8 in the culture medium of NHDF (35,000 cells/cm^2^), treated with NFs for 24 h and, then, with lipopolysaccharide (LPS) at the concentration of 10 μg/mL for another 24 h, were detected by means of Human IL-8/CXCL8 ELISA kit (Sigma Aldrich, St. Louis, MO, USA) according to the manufacturer's instructions. Untreated (without samples) cells subjected to LPS-inflammation were considered as the negative control (LPS), while untreated cells not subjected to LPS-inflammation (CM) was used as reference. An Alamar Blue assay was also performed in order to evaluate if the inflammation could alter cell viability %; three replicates were performed for each sample.

#### 2.2.7 Preparation of the EC/SG mucoadhesive film

Films were prepared by the solvent-casting method. Briefly, ethyl cellulose (EC) was dissolved in absolute ethanol at a concentration of 2% w/v under continuous magnetic stirring until complete polymer solubilization was reached [22]. Stock solutions of SG60 and Sangelose® 90L (SG90) were individually prepared at 1.5% w/v in ultrapure water, following the procedure described in Section 2.2.3. Each SG solution was then separately mixed with the EC solution at EC/SG volume ratios of 75:25, 65:35 and 55:45 (% v/v), hereafter referred to as EC/SG 1, EC/SG 2 and EC/SG 3, respectively, to obtain EC/SG60 and EC/SG90 mixtures. Polyethylene glycol (PEG) was incorporated at a concentration of 2.5% w/v into each formulation as a plasticiser. The resulting film-forming solutions were cast into glass Petri dishes and allowed to dry at room temperature until solvent evaporation was complete. The dried films were carefully detached and stored in a desiccator over silica gel until further use.

#### 2.2.8 Film characterizations

##### 2.2.8.1 Mucoadhesive properties

The mucoadhesive behaviour of the EC/SG systems was evaluated using a TA.XT plus Texture Analyser (Stable Micro Systems, UK) equipped with a 1 kg load cell, a cylindrical probe (P/10, ø = 10 mm), and a dedicated Mucoadhesion Rig. The apparatus consisted of two vertically aligned cylinders: the bottom one with a flat platform and the upper one featuring a central opening through which the probe could move. The entire setup was maintained at 37 °C by immersing the rig in a temperature-controlled water bath to mimic physiological conditions. A 0.5% w/v porcine gastric mucin dispersion (Sigma-Aldrich, Italy) prepared in SSF served as the biological substrate, while SSF alone was used for blank measurements. A 200 μL aliquot of either mucin dispersion or SSF was placed onto a filter paper disc fixed on the lower cylinder. The probe, covered with a bi-adhesive tape and a 10 mm diameter filter paper, was loaded with 50 mg of the EC/SG formulations. During testing, the probe moved downward at 1.00 mm/s until contacting the mucin or SSF layer, applying a preload force of 2.5 N for 60 s. It was then retracted at 0.50 mm/s until complete separation of the sample from the substrate occurred. Each condition (with and without mucin) was assessed in six replicates. The maximum detachment force (F_Max_, N) corresponding to the peak of the force–distance curve, represents the maximum force required to separate the formulation from the substrate and was used as an indicator of mucoadhesive strength.

##### 2.2.8.2 Mechanical properties

Mechanical properties of EC/SG systems were evaluated using a Texture Analyzer, according to the procedures described in Section 2.2.6.3. For EC/SG films, Young's modulus was calculated from the common linear region of the stress–strain curves (25–45% strain).

#### 2.2.9 Coating of NAR-NCs_NFs with mucoadhesive films

NAR-NCs_NFs were coated with EC/SG films using the following procedure. A volume of 2.5 mL of EC/SG mixture was poured into dedicated glass Petri dishes and left under a fume hood for partial solvent evaporation, yielding a semi-solid base layer. Subsequently, a NAR-NCs_NFs mat sample (1.5 cm × 1.5 cm) was carefully placed onto each semi-solid layer. An additional 2.5 mL of the same polymeric mixture was then dispensed over the fibres to ensure complete coverage. The coated systems were left under the fume hood for 24 h to allow solvent evaporation. The obtained EC/SG coated NFs were detached from the Petri dishes and stored in a desiccator over silica gel until further analyses. The mucoadhesive properties of the coated systems were evaluated using the Texture Analyzer according to the procedure described in Section 2.2.8.1. The coated systems were further characterized for their mechanical properties using a Texture Analyzer, according to the procedures described in Section 2.2.6.3. Young's modulus was calculated from the common linear region of the stress–strain curves (25–45% strain). The coated systems were subsequently characterised for their *in vitro* drug-release behaviour using the method described in Section 2.2.6.4.

Finally, preliminary cytocompatibility studies of EC/SG_NFs were carried out *in vitro* using NHDF as reported in Section 2.2.6.5. In addition to acute cytocompatibility assessment, a proliferation assay was performed to assess cell growth under continuous extract exposure over a 7-day period. NHDF cells were seeded in 96-well plates at a density of 12,500 cells/well in 100 μL of complete medium (CM). Simultaneously, 100 μL of either CM or 2-fold concentrated EC/SG_NFs extract (prepared at 20 mg/mL) was added to each well, resulting in a final extract concentration of 10 mg/mL and ensuring continuous exposure from the time of seeding. Cell proliferation was monitored over 7 days, with viability assessments conducted at days 1, 3, and 7 using the Alamar Blue assay.

#### 2.2.10 Statistical analysis

Statistical analyses were performed using GraphPad Prism (version 10.1.1, GraphPad Software, USA). One-way ANOVA followed by Tukey's post hoc test or unpaired t-tests were applied, as appropriate.

## 3 Results

### 3.1 Naringenin-nanocrystals

Wet media milling is a well-established top-down approach for producing drug NCs, in which hard beads transmit collision, attrition and shear forces to the drug suspension. A distinctive feature of this technique compared to conventional dry milling is the presence of a liquid phase containing stabilizers (e.g., polymers or surfactants), which not only prevent particle agglomeration during size reduction but also ensure long-term colloidal stability of the resulting dispersion. Conventional milling, including both dry methods (e.g., jet milling) and classical wet media milling with stirred bead mills, often requires extended processing times - ranging from tens of minutes to hours - to obtain stable NCs with optimal size and low polydispersity, limiting its applicability for rapid formulation screening. DAC, also known as “speed-mix” technology, overcomes this limitation by rotating the sample simultaneously around its own axis and a central axis, generating intense shear and mixing. This enables efficient particle size reduction in significantly shorter times. P188, a non-ionic surfactant commonly used in NCs formulations, was employed as a stabilizer to prevent particle aggregation and improve colloidal stability. The combined use of DAC milling and P188 has recently demonstrated the ability to produce NCs of poorly soluble drugs within minutes, offering a fast, efficient and reproducible platform for early-stage formulation development [19].

**Fig. 1:**
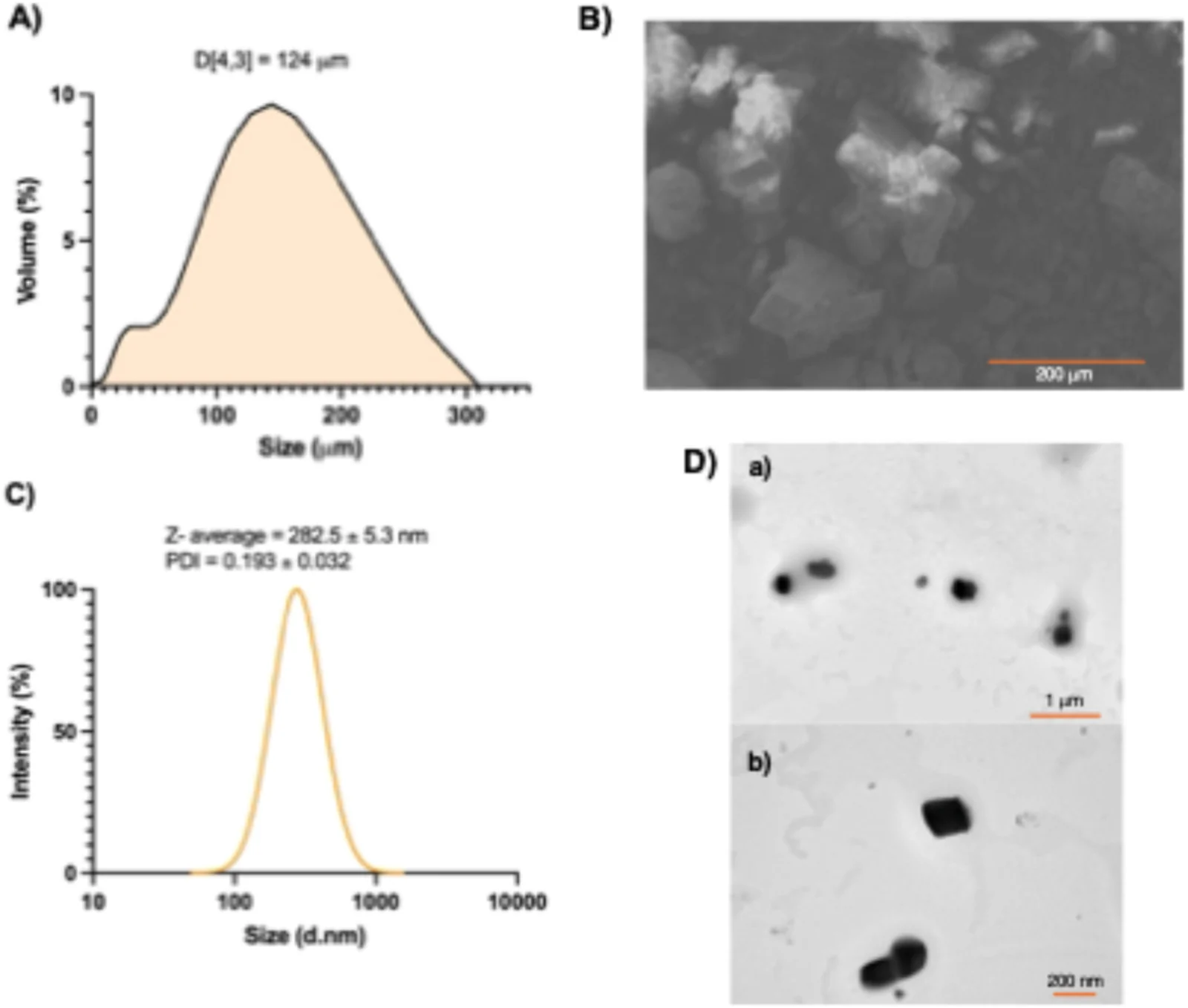
Morphological and physicochemical characterisation of raw naringenin (NAR) and naringenin nanocrystals (NAR-NCs). (A) Particle size distribution of raw NAR determined by laser diffraction. (B) Scanning electron microscopy (SEM) micrograph of crude NAR (500×). (C) Hydrodynamic size distribution of NAR-NCs determined by dynamic light scattering (DLS). (D) Transmission electron microscopy (TEM) micrographs of NAR-NCs acquired from different representative fields of view at (a) 10,000× and (b) 30,000×. Panel (a) shows the morphology of multiple nanocrystals, while panel (b) presents representative individual nanocrystals at higher magnification.

Prior to milling, the particle size of raw NAR powder was assessed to establish a baseline for size reduction. The data revealed a broad size distribution, covering an interval of approximately 300 μm, with a De Brouckere mean diameter (D [3,4]) equal to 124 μm (Fig. 1A). SEM imaging confirmed a heterogeneous morphology, showing large and angular particles prone to aggregation (Fig. 1B). After wet milling, well-dispersed NAR-NCs were obtained, exhibiting a mean hydrodynamic diameter of 282.5 ± 5.3 nm and a polydispersity index (PDI) of 0.193 ± 0.032, indicative of a narrow, monomodal distribution suitable for NCs-based formulations. DLS analysis confirmed this, showing a Gaussian-like intensity profile peaking at ∼280–300 nm and a single, well-defined population (Fig. 1C).

TEM images revealed that the NAR-NCs exhibited a darker core, representing the drug particles, surrounded by a lighter layer attributed to the stabilizer P188 (Fig. 1D). The particle size measured by TEM was slightly smaller (∼210 nm), consistent with the expected differences between the techniques: TEM provides a direct measurement in the solid state, whereas DLS measures the hydrodynamic diameter in dispersion, which can be influenced by solvent interactions, swelling, and the stabilizer layer [23,24].

A 10-day stability study at room temperature showed a modest increase in particle size to 303.3 ± 4.0 nm (mean ± SD n = 3), with no significant changes in PDI values (0.176 ± 0.02; mean ± SD n = 3), remaining within the typical range for stable drug NCs (100–1000 nm) [25]. Redispersion after centrifugation confirmed the absence of irreversible aggregation, with only slight increase in particle size immediately after redispersion (303.3 ± 2.1 nm; PDI 0.18 ± 0.02; mean ± SD n = 3) and after 10 days (343.6 ± 5.0 nm; PDI 0.20 ± 0.02; mean ± SD n = 3), still consistent with stable NCs.

Wet media milling can induce structural modifications in drug particles, such as partial amorphization or polymorphic transitions, particularly when the applied mechanical stress exceeds a critical threshold. The extent of these alterations largely depends on the intrinsic physicochemical properties of the drug, the nature of the stabilizer used, and the specific milling parameters [13]. To assess whether the crystalline structure of NAR was preserved after milling, DSC, TGA, and FT-IR analyses were carried out on NAR-NCs, their individual components (raw NAR and P188), and the corresponding PM.

**Fig. 2:**
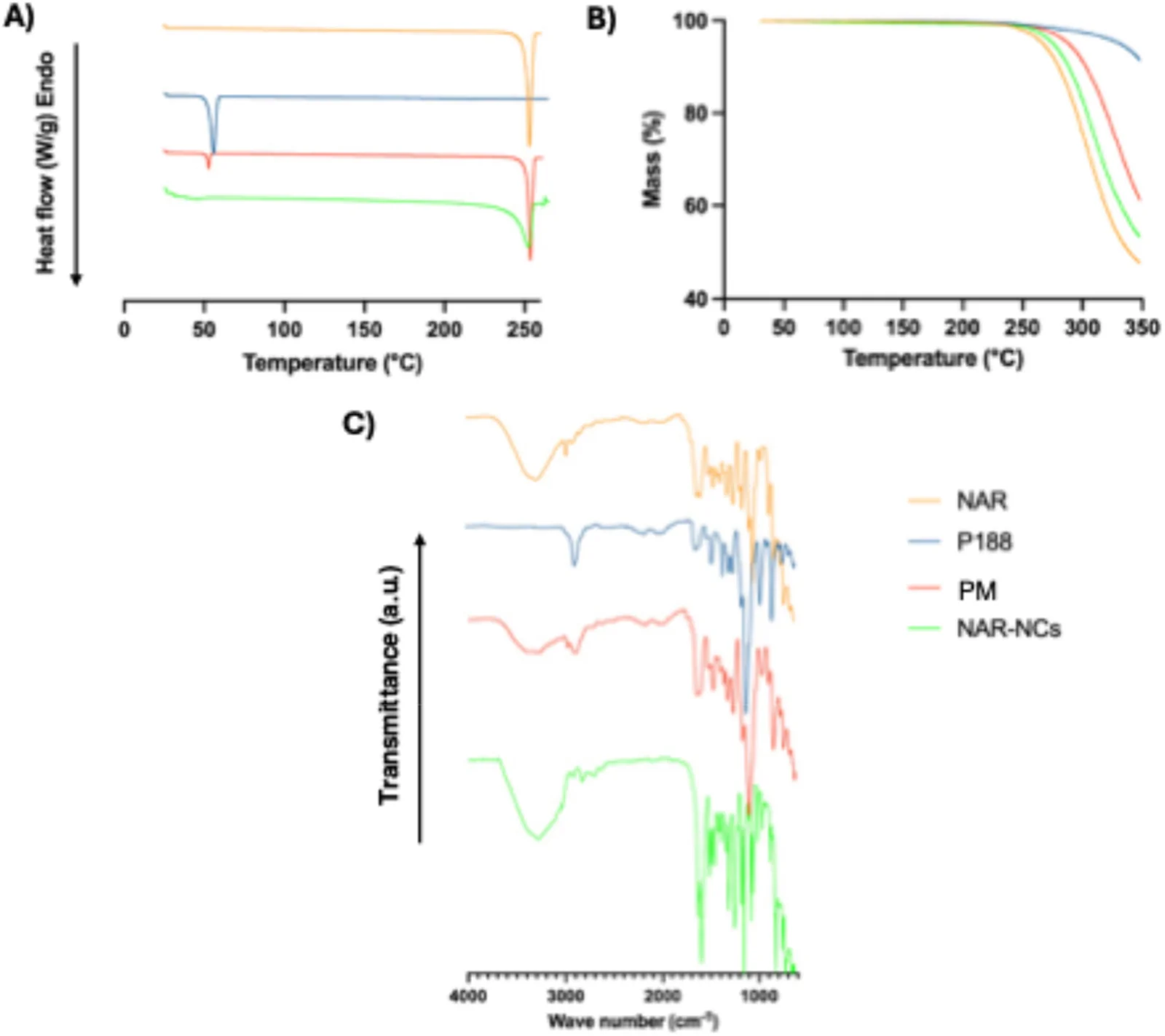
Solid-state characterisation of raw naringenin (NAR), poloxamer 188 (P188), the corresponding physical mixture (PM), and naringenin nanocrystals (NAR-NCs). (A) Differential scanning calorimetry (DSC) thermograms. (B) Thermogravimetric analysis (TGA) curves showing the residual mass (%) as a function of temperature. (C) Fourier-transform infrared (FT-IR) spectra. FT-IR transmittance is presented in arbitrary units (a.u.).

DSC was used to detect possible interactions between the drug and excipients and to monitor changes in drug crystallinity. As shown in Fig. 2A, raw NAR exhibited a sharp endothermic melting peak at ∼253 °C, which was also observed in both PM and NAR-NCs, confirming the preservation of the NAR crystalline form. Notably, a slight shift of the melting peak in NAR-NCs to lower temperatures was evidenced, likely due to the reduced particle size and increased surface energy, in line with the Gibbs–Thomson effect [13]. The characteristic melting peak of P188 at ∼55 °C was not distinctly visible in NAR-NCs, probably due to the removal of excess stabilizer during centrifugation [26]. TGA thermograms (Fig. 2B) demonstrated that all samples were thermally stable up to ∼250 °C. NAR-NCs showed a slight delayed onset of decomposition compared to the raw drug, suggesting that the milling process did not compromise thermal stability or induce significant degradation.

The FT-IR spectrum of raw NAR (Fig. 2C) exhibited characteristic absorption bands corresponding to its functional groups. A broad band at ∼3280–3290 cm^−1^ corresponded to the stretching vibration of phenolic –OH groups, while a signal at around 3050–3115 cm^−1^ was attributed to aromatic C–H stretching. A strong band at ∼1625–1630 cm^−1^ was assigned to C=O stretching of the flavanone backbone, accompanied by bands at ∼1600 cm^−1^ indicative of aromatic C=C stretching. Further characteristic peaks were observed at ∼1460 cm^−1^ (CH_2_ bending), ∼1385 cm^−1^ (CH_3_ bending), and ∼1010 cm^−1^ (C–O stretching). These signals are consistent with previously reported spectra of naringenin and confirm its structural integrity and crystalline nature [27,28]. The FT-IR spectra of both the PM and NAR-NCs (Fig. 2C) displayed peaks similar in position and shape to those of raw NAR, indicating that drug functional groups remained unchanged during processing. The characteristic bands of P188 were detected in the PM, particularly at 2875 cm^−1^ (C–H stretching), 1341 cm^−1^ (O–H bending), and 1094 cm^−1^ (C–O stretching) [15]. In contrast, these peaks appeared markedly reduced or absent in NAR-NCs spectrum, most likely due to partial removal of excess stabilizer during centrifugation. Importantly, no new peaks or significant shifts were observed in NAR-NCs spectrum, confirming the absence of chemical interactions between NAR and P188. These findings are in agreement with DSC and TGA results, supporting the preservation of NAR crystallinity and the physical rather than chemical nature of drug–stabilizer interactions.

Following solid-state characterization, the drug content of NAR-NCs was quantified spectrophotometrically. The drug content of NAR-NCs, expressed as the percentage of NAR recovered in the NCs pellet relative to the theoretical amount, was 72.5 ± 2.3% (mean ± SD n = 3). After centrifugation, this value slightly decreased to 65.2 ± 2.6% (mean ± SD n = 3), likely reflecting partial loss of NAR during the centrifugation and resuspension steps. This trend is consistent with previous findings on poorly soluble drug NCs, including the work of Dong et al. [29]. After determining the drug content, the saturation solubility in water was then evaluated. Raw NAR showed low solubility, equal to 47.1 ± 0.1 μg/mL (mean ± SD n = 3), while the PM reached 73.2 ± 1.1 μg/mL (mean ± SD n = 3). In contrast, NAR-NCs exhibited a significantly higher solubility of 171.1 ± 1.0 μg/mL (mean ± SD n = 3), corresponding to a 3.6-fold increase compared to the raw drug, achieved after just 18 min of milling. This improvement is primarily attributed to the nanoscale size of the drug crystals, which increases the specific surface area and dissolution rate in accordance with the Noyes–Whitney equation. In addition, the higher particle curvature enhances dissolution pressure, allowing more molecules to remain in solution, in line with the Ostwald–Freundlich effect [13]. P188 also contributes by stabilizing the nanosuspension and limiting particle aggregation. These results align with previous studies demonstrating the ability of nanonization to significantly improve the solubility and dissolution rate of poorly water-soluble drugs [30,31]. Overall, the DAC-mediated wet milling process efficiently produced NAR-NCs with preserved crystallinity, high drug content, and markedly enhanced aqueous solubility, supporting NCs potential as a robust and reproducible platform for further formulation development.

### 3.2 Core-shell nanofibres

To exploit the advantages of NAR-NCs for buccal delivery, NAR-NCs were incorporated into polymeric NFs produced via electrospinning. Electrospinning is a versatile and scalable technique for fabricating polymer-based fibres ranging from the micro-to nanoscale range. A typical setup includes a syringe with a metallic needle, a syringe pump, a high-voltage power supply, and a grounded collector. Upon application of high voltage, the polymer solution at the needle tip elongates into a conical structure, known as the Taylor cone, from which a continuous jet is ejected and solidifies into fibres on the collector. The stability of the Taylor cone is crucial for achieving uniform fibre morphology [32,33]. In coaxial electrospinning, two concentrically aligned needles allow for the simultaneous extrusion of core and shell solutions, enabling the fabrication of core-shell fibres with advanced functionalities, such as drug encapsulation and controlled release. Core–shell NFs have recently attracted increasing attention for the sustained delivery of drugs, proteins, and genes, as they can reduce burst release, prolong release over time, and preserve the activity of labile molecules. Their architecture also allows the use of unspinnable solutions in the core, the co-loading of multiple agents with tuneable release profiles, and the protection of the encapsulated ingredients by the shell, ultimately improving therapeutic efficacy and minimizing toxicity [17,34,35].

The hydrophilic core was designed using PVA blended with SG60, a hydrophobically modified hydroxypropyl methylcellulose containing stearoyl substitutions on the hydroxypropyl moiety. This polymeric matrix was selected to stabilize the encapsulated NAR-NCs and minimize particle aggregation during storage. The shell consisted of PCL, a hydrophobic biodegradable polymer widely used in biomedical applications, providing a diffusion barrier for sustained drug release [36,37].

**Fig. 3:**
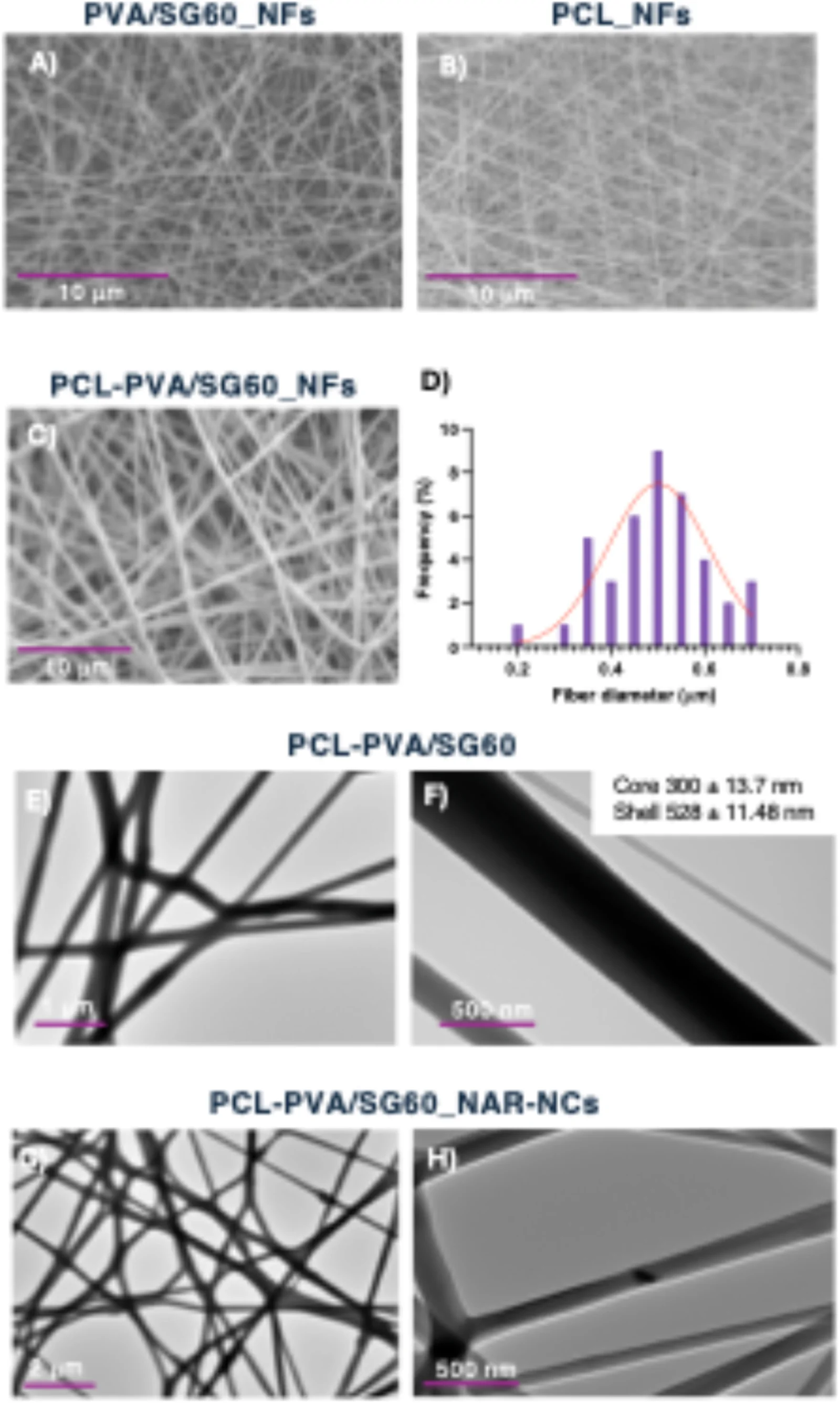
Morphological characterisation of the electrospun nanofibres. Scanning electron microscopy (SEM) micrographs of PVA/SG60 nanofibres (NFs) (A), polycaprolactone (PCL) nanofibres (B), and coaxial PCL–PVA/SG60 core–shell nanofibres (C). (D) Fibre diameter distribution of the coaxial nanofibres determined from 50 measurements using ImageJ™ (mean ± SD); the red curve represents the fitted distribution. Transmission electron microscopy (TEM) micrographs of the coaxial PCL–PVA/SG60 nanofibres acquired at 15,000× (E) and 30,000× (F), confirming the formation of the core–shell structure. TEM micrographs of PCL–PVA/SG60 nanofibres loaded with naringenin nanocrystals (PCL–PVA/SG60_NAR-NCs) acquired at 5000× (G) and 15,000× (H), demonstrating the successful incorporation of NAR-NCs within the nanofibrous system.

The first stage of characterization focused on evaluating NF morphology by SEM and TEM imaging to confirm fibre uniformity and the successful formation of the core–shell architecture (Fig. 3). SEM analysis was first performed to investigate the surface morphology of the individual core (PVA/SG60) and shell (PCL) NFs. As shown in Fig. 3A and B, both core and shell NFs exhibited smooth and uniform surfaces, with no evidence of bead formation, indicating optimal electrospinning conditions. Under coaxial electrospinning, the resulting core–shell NFs also showed a uniform and bead-free morphology, with a mean diameter of 540 ± 12.5 nm (n = 100). The diameter distribution, calculated from 100 randomly selected fibres (Fig. 3D), followed a Gaussian profile and yielded a mean value of 540 ± 56 nm, confirming the homogeneity of the fibre population. Further insights into the internal architecture of core-shell NFs were provided by TEM (Fig. 3E and F), which clearly confirmed the formation of a distinct core–shell structure. The mean diameters measured for the core and shell NFs were 300 ± 13.7 nm and 528 ± 11.5 nm, respectively, consistent with the successful encapsulation of NAR-NCs within the hydrophilic core matrix. Following the fabrication of empty NFs, attention was turned to the encapsulation of NAR-NCs within the PVA/SG60 core. TEM analysis of NAR-NCs-loaded fibres (Fig. 3G and H) clearly confirmed successful incorporation, with NCs visibly incorporated within the fibre core. The fibres retained a generally uniform and bead-free morphology; however, the diameter distribution was slightly broader compared to that of the empty fibres, with a mean diameter of 537 ± 67 nm. This modest increase in size variability is likely attributable to the presence of NAR-NCs within the core, which may affect jet stability and fibre formation during the coaxial electrospinning process.

A critical parameter for NAR-NCs is their ability to redisperse into the original nanometric size range after the encapsulation into a solid formulation, as it directly influences the preservation of their key advantages, namely enhanced dissolution rate and increased saturation solubility [38]. To assess the impact of electrospinning on NC size, DLS measurements were performed on the NAR-NCs dispersion after centrifugation (NAR-NCs_pellet) and on NAR-NCs dispersion after fibre core solubilization (NAR-NCs_core). The hydrodynamic diameter increased slightly from 303.3 ± 2.1 nm for NAR-NCs_pellet to 357 ± 3.2 nm for NAR-NCs_core (p < 0.0001), whereas no significant differences were observed in PDI (0.18 ± 0.02 and 0.19 ± 0.04, respectively). Direct measurement of NAR-NCs incorporated into core-shell NFs mats was not pursued, due to the insolubility of the PCL shell in water, which hinders complete core-shell dissolution and may lead to detect fibre fragments or aggregates rather than free NAR-NCs. The observed increase of approximately 50 nm in hydrodynamic diameter is likely attributable to interactions between the polymeric matrix and the P188 layer surrounding the NAR-NCs during the electrospinning and redispersion processes, consistent with previous reports by Zhou et al. and Zhang et al. [15,39]. Nevertheless, the recovered NAR-NCs remained within the 200–500 nm size range considered optimal for buccal delivery.

Following confirmation that the electrospinning process preserved the nanometric size of NAR-NCs, the drug content of NAR-NCs_NFs was determined to be 0.047 ± 0.005 mg/cm^2^, suggesting a homogeneous distribution of the nanocrystals within the fibrous matrix.

**Fig. 4:**
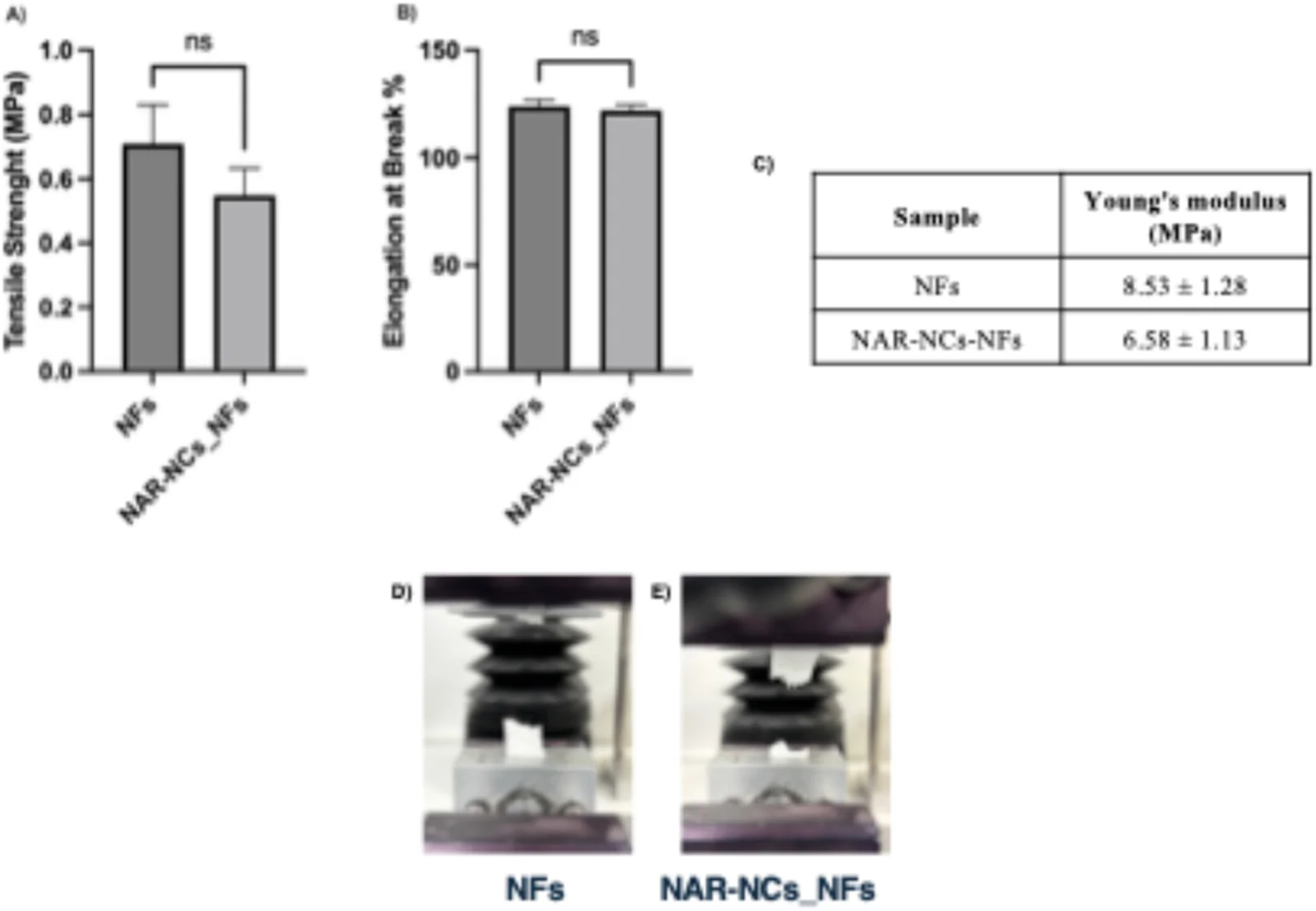
Mechanical characterisation of unloaded nanofibres (NFs) and naringenin nanocrystal-loaded nanofibres (NAR-NCs_NFs). (A) Tensile strength and (B) Elongation at Break (%) of the electrospun nanofibres. (C) Young's modulus values determined from the initial linear elastic region (2–8% strain) of the corresponding stress–strain curves. Data are presented as mean ± SD (n = 3). Statistical analysis was performed using an unpaired two-tailed *t*-test. Representative photographs acquired during tensile testing with the Texture Analyzer showing the breaking point of unloaded NFs (D) and NAR-NCs_NFs (E).

Tensile properties of both unloaded NFs and NAR-NCs_NFs were evaluated and are reported in Fig. 4, in terms of maximum tensile strength (A) and elongation at break (B), while representative stress–strain curves are shown in Fig. S1A. No statistically significant differences were observed between the two formulations for all parameters, indicating that the incorporation of NAR-NCs did not alter either the overall mechanical performance or the initial stiffness of the electrospun nanofibres. These findings demonstrate that embedding the nanocrystals within the polymeric core preserves the structural integrity, flexibility and elastic behaviour of the fibrous matrix. Such characteristics are particularly desirable for buccal applications, where the dressing should combine sufficient mechanical robustness with the ability to conform to the dynamic movements of the oral mucosa. Moreover, the tensile strength values obtained are in line with those reported for chitosan-based buccal films incorporating microspheres for peptide delivery (≈0.7 MPa) [40], further supporting the suitability of the developed system for buccal drug delivery.

**Fig. 5:**
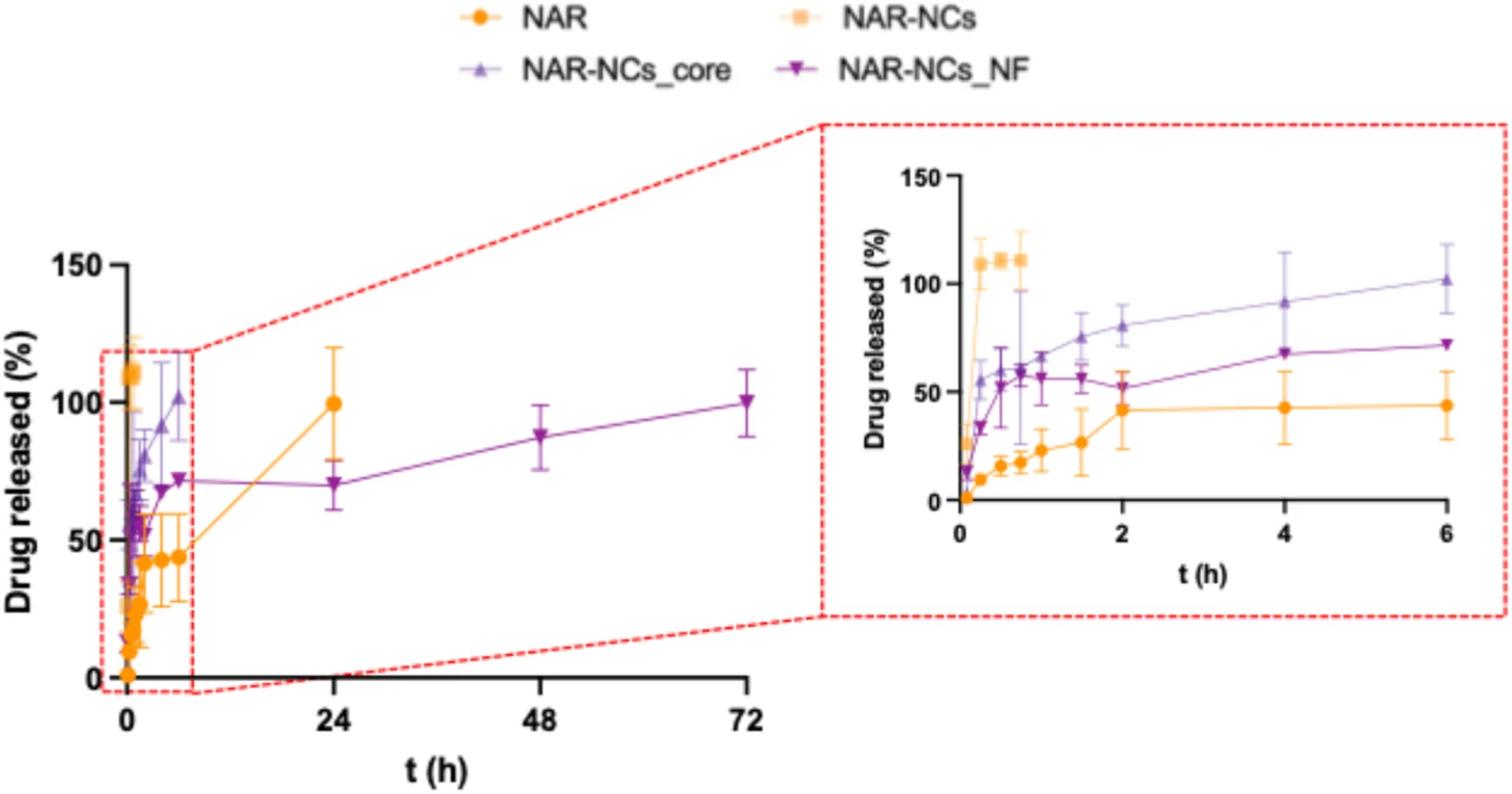
In vitro release profiles of raw naringenin (NAR), naringenin nanocrystals (NAR-NCs), NAR-NCs recovered after dissolution of the fibre core (NAR-NCs_core), and NAR-NCs-loaded core–shell nanofibres (NAR-NCs_NFs) in simulated saliva fluid (SSF). The inset shows an enlarged view of the initial release phase (0–6 h) to facilitate comparison of the early release behaviour among the different formulations. Data are presented as mean ± SD (n = 3).

The *in vitro* release profiles of raw NAR, NAR-NCs, NAR-NCs-loaded core NFs and NAR-NCs_NFs in SSF are reported in Fig. 5. The saturation solubility of raw NAR and the different NAR-containing formulations was determined in SSF containing 2% (w/v) Tween 80 to establish the release conditions. Raw NAR exhibited a saturation solubility of 0.113 ± 0.01 mg/mL (mean ± SD n = 3). The corresponding saturation solubility values for NAR-NCs, NAR-NCs_core and NAR-NCs_NFs were 0.938 ± 0.1, 0.641 ± 0.06 and 0.645 ± 0.05 mg/mL (mean ± SD n = 3), respectively. These values were used to select an appropriate dissolution-medium volume and ensure sink conditions throughout the *in vitro* release study. As expected, raw NAR exhibited minimal dissolution, reaching its saturation solubility only after ∼24 h, and thus confirming its poor aqueous solubility and intrinsically slow dissolution kinetics. In contrast, NAR-NCs demonstrated significantly enhanced dissolution performance, with nearly complete release (∼100%) achieved within 45 min. This behaviour is consistent with the increased saturation solubility observed for the NCs and highlights the impact of nanonization in accelerating drug dissolution compared to raw NAR. The NAR-NCs-loaded core (NAR-NCs_core) fibres exhibited a distinct release profile, with complete drug release occurring after approximately 6 h. This slower release suggests that the polymeric matrix modulates NAR availability, providing a controlled release. The delayed profile is likely attributable to the hydration and gradual erosion of the relatively thick PVA/SG60 matrix, which controls the rate at which NAR becomes available to the dissolution medium. Finally, NAR-NCs_NFs exhibited the most prolonged release profile, with complete drug release extending up to 72 h. This long-acting release highlights the capacity of electrospun nanofibres to sustain drug delivery over time. The polymeric architecture, combined with the distribution of NAR-NCs within the fibrous core, likely controls the rate at which NAR becomes available to the external medium, resulting in the observed prolonged release profile. Such a profile is particularly advantageous for buccal drug delivery, where prolonged residence time and sustained local drug availability are desirable. The prolonged-release behaviour of the electrospun nanofibres, combined with the enhanced saturation solubility and dissolution rate provided by NAR-NCs, could help to maintain a continuous source of dissolved NAR at the mucosal surface over time.

Taken together, these findings demonstrate that the incorporation of NCs into electrospun fibres enables the release of otherwise poorly soluble NAR, while allowing its release profile to be tailored for buccal delivery.

#### 3.2.1 Cytocompatibility

**Fig. 6:**
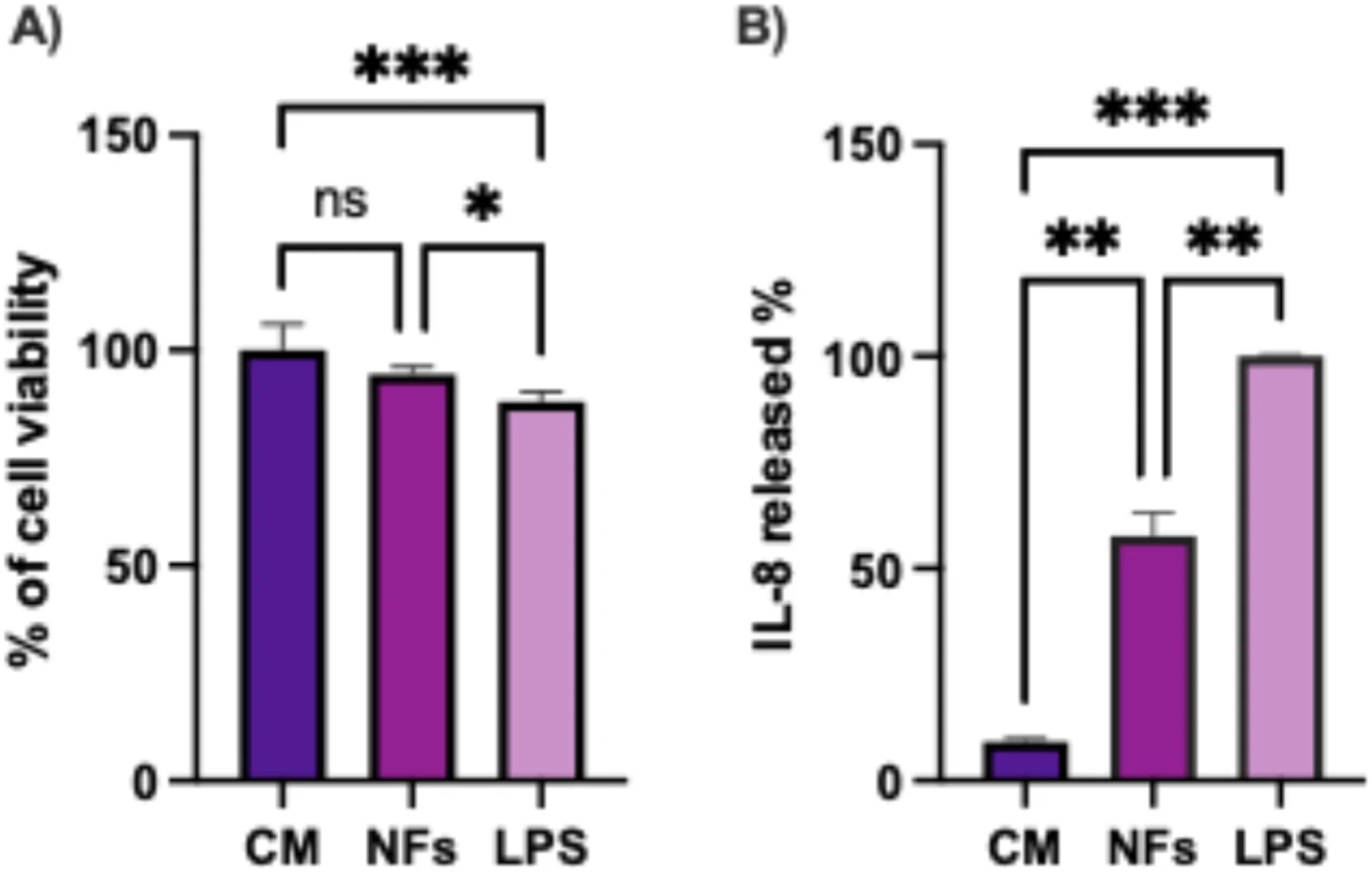
**–** (A) Cell viability and (B) IL-8 release following pre-treatment with the NFs extract, diluted 1:1 v/v in complete medium (CM), for 24 h and subsequent stimulation with LPS (10 μg/mL) for a further 24 h. Cell viability values were normalised to untreated cells maintained in CM, which were set at 100%. IL-8 release values were normalised to LPS-stimulated cells not pre-treated with the NFs extract, which were set at 100%. Data are presented as mean ± SD (n = 6). One-way ANOVA followed by Tukey's multiple comparisons test (*p < 0.05, **p < 0.01, ***p < 0.001).

The cytocompatibility of the developed NFs was assessed using normal human dermal fibroblasts (NHDF), which were selected as a primary human cell model to evaluate the cytocompatibility and anti-inflammatory activity of the developed nanofibrous platform. This approach is consistent with previous studies on formulations intended for oral mucosal applications, including oral mucositis, where human dermal fibroblasts have been employed to investigate cytocompatibility, cell proliferation, and inflammatory responses [41,42]. Under the experimental conditions used for the anti-inflammatory assay, cell viability remained above the 70% threshold defined by ISO 10993-5 for non-cytotoxic materials (Fig. 6A) [43]. These findings indicate that the developed NFs did not adversely affect cell viability, supporting the favourable cytocompatibility profile of the platform for local buccal application.

#### 3.2.2 Anti-inflammatory activity

To investigate whether the formulation preserved the biological activity of encapsulated NAR, the anti-inflammatory potential of the NFs extract was evaluated under LPS-induced inflammatory conditions. As shown in Fig. 6A, LPS stimulation caused a slight reduction in NHDF viability compared with untreated cells (CM). Nevertheless, cell viability remained above 80% under all tested conditions, confirming that the observed changes in IL-8 release could be evaluated independently of marked cell death.

The anti-inflammatory response was further assessed by measuring IL-8 release, a key pro-inflammatory cytokine involved in the development and progression of oral mucositis. As shown in Fig. 6B, pre-treatment with the NFs extract significantly reduced IL-8 secretion compared with LPS-stimulated cells not pre-treated with the NFs extract, indicating attenuation of the inflammatory response. IL-8 plays a central role in the pathogenesis of oral mucositis by promoting inflammatory cell recruitment and amplifying local tissue inflammation [44,45]. Taken together, these findings suggest that NAR retained its biological activity following nanocrystal production and encapsulation within the electrospun nanofibres. Importantly, the preservation of this anti-inflammatory response indicates that neither wet media milling nor electrospinning compromised the biological activity of NAR, consistent with previous reports describing its inhibitory effects on pro-inflammatory cytokine production and inflammatory signalling pathways [46].

### 3.3 EC/SG mucoadhesive film coating

Building on the results obtained with the NAR-NCs_NFs, a mucoadhesive film was developed to offer a patient-friendly dosage form with prolonged residence time on the buccal mucosa. Films were produced by solvent casting using SG and EC as the main polymeric components. Two SG grades (SG60L and SG90L) were selected for their amphiphilic structure and well-documented mucoadhesive properties [47]. Although EC is not inherently mucoadhesive, it slows hydration and dissolution, thereby contributing to extended residence time. Upon contact with biological fluids, the films are expected to hydrate and swell, forming thin hydrogel-like layers capable of adhering to mucosal surfaces and retaining the core–shell fibres *in situ*.

**Fig. 7:**
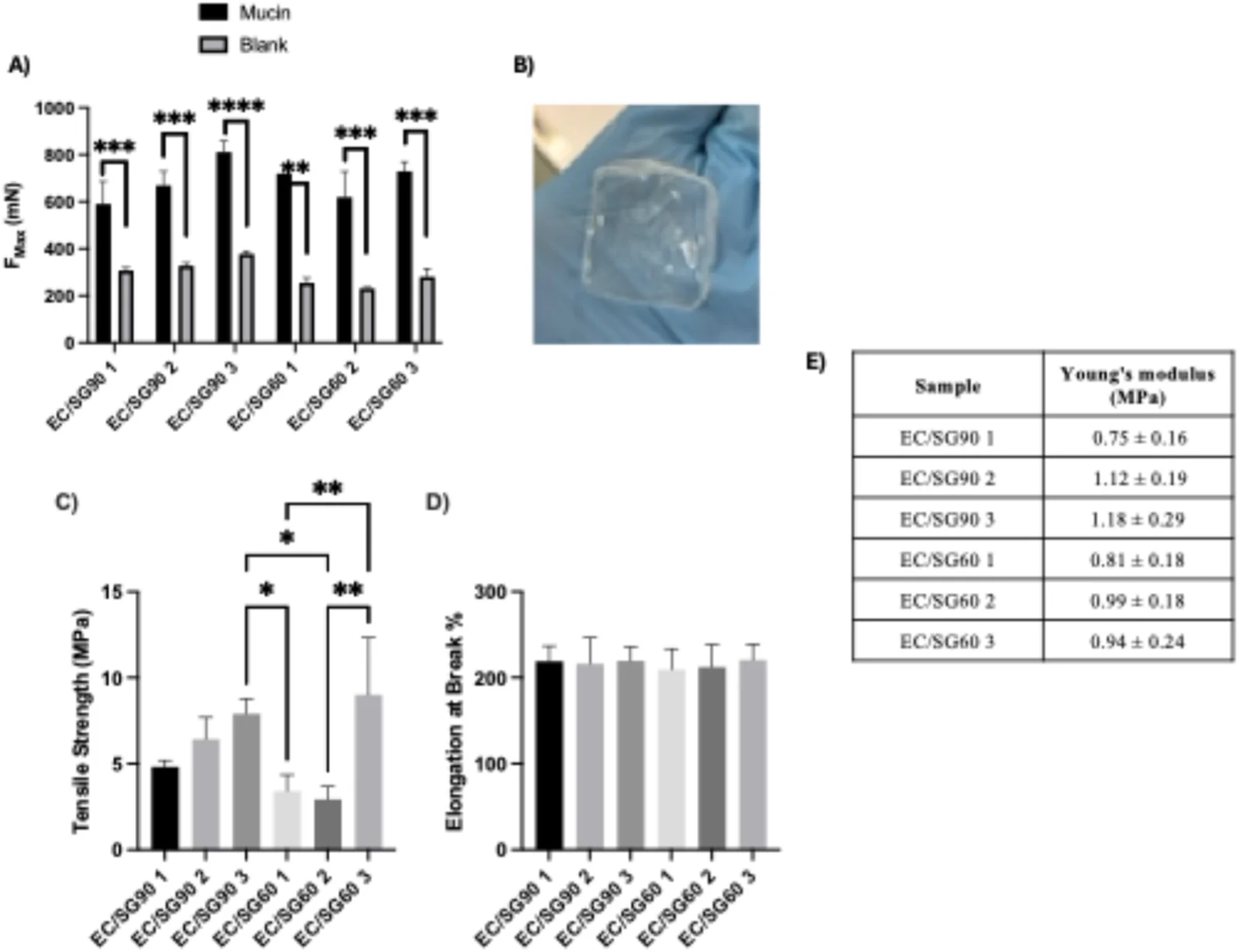
Characterisation of the EC/SG mucoadhesive films. (A) Maximum detachment force (F_Max_) measured after 60 s of contact with mucin or in its absence (blank) at 37 °C for EC/SG60 and EC/SG90 films prepared using three EC/SG volume ratios: EC/SG 1 (75:25), EC/SG 2 (65:35) and EC/SG 3 (55:45) (% v/v). (B) Representative photograph of an EC/SG film. (C) Tensile strength and (D) Elongation at Break % of the EC/SG films. (E) Young's modulus values determined from the slope of the common linear region (25–45% strain) of the corresponding stress–strain curves. Data are presented as mean ± SE (n = 4). Statistical analysis was performed using one-way ANOVA followed by Tukey's multiple comparisons test (*p < 0.05, **p < 0.01, ***p < 0.001, ****p < 0.0001).

The mucoadhesive performance of the EC/SG films is shown in Fig. 7A. For all formulations, F_Max_ values in the presence of mucin were markedly higher than under blank conditions, confirming effective interaction with the mucus substrate. This behaviour is consistent with the known mucoadhesive mechanism of SG, which establishes hydrogen bonds and physical entanglements with mucin glycoproteins [48,49]. Overall, F_Max_ ranged from approximately 600 to 900 mN, values that exceed the levels typically regarded as sufficient for mucosal bioadhesion. Indeed, Bazan et al. and Olechno et al. reported that detachment forces of 300-500 mN generally provide adequate adhesion for mucosal delivery systems [50,51]. The higher F_Max_ measured for EC/SG films therefore indicates robust mucoadhesive capacity, suggesting the ability to maintain prolonged residence on the buccal mucosa while ensuring stable retention of the core–shell nanofibres. A representative photograph of an EC/SG film is provided in Fig. 7B.

Fig. 7C–E and Fig. S3B present the mechanical properties of the EC/SG films in terms of tensile strength, elongation at break and Young's modulus and representative stress–strain curves, respectively. An overall reinforcement effect was observed, with the formulations containing the highest SG content (EC/SG90 3 and EC/SG60 3) exhibiting significantly higher tensile strength than their lower-SG counterparts. This behaviour is consistent with the greater polymer–polymer entanglement and intermolecular interactions introduced by SG, which lead to a more cohesive network. Importantly, this increased tensile strength did not compromise flexibility. Elongation at break consistently remained around 200% for all formulations, indicating that the films retain high deformability without becoming brittle, an essential requirement for buccal patches, which must withstand manipulation and oral movements.

Young's modulus values (Fig. 7E) ranged from 0.75 ± 0.16 to 1.18 ± 0.29 MPa. Only modest variations were observed among the different formulations, suggesting that the initial linear mechanical response of the films was largely preserved across the investigated EC/SG ratios.

Overall, EC/SG films displayed tensile strengths of ∼3–9 MPa, elongation at break of ∼200% and Young's modulus values ranging from 0.75 to 1.18 MPa, all of which fall within the range commonly reported for mucoadhesive buccal films. Previous studies on intraoral polymeric blends describe tensile strengths between ∼2.33 and ∼12.01 MPa, depending on polymer composition, confirming that the EC/SG systems achieve mechanical performance comparable to established buccal platforms.

**Fig. 8:**
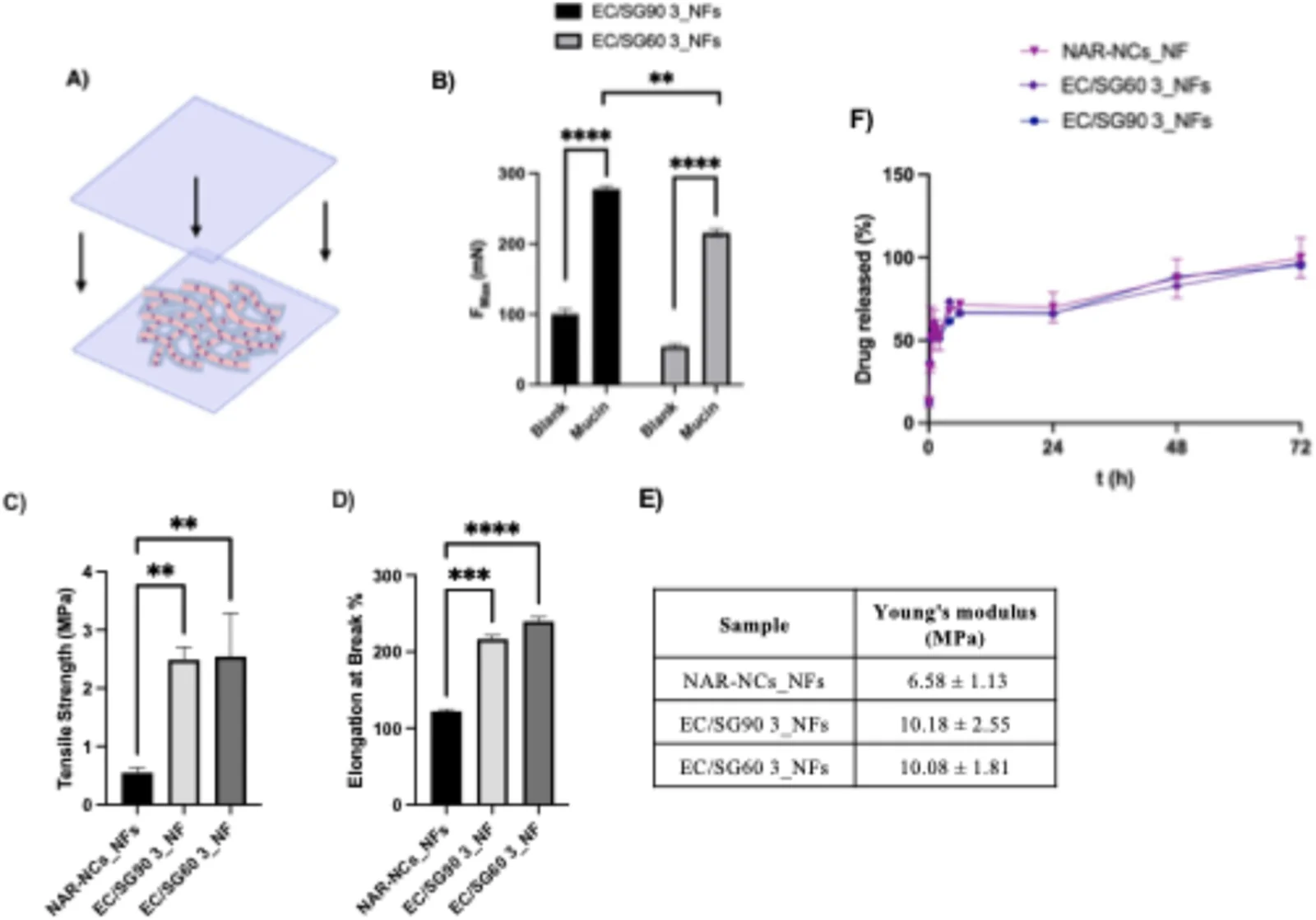
Characterisation of the integrated EC/SG_NF platform. (A) Schematic illustration of the EC/SG_NF system, consisting of the selected EC/SG film laminated onto the NAR-NCs-loaded core–shell nanofibres. (B) Maximum detachment force (F_Max_ measured after 60 s of contact with mucin or in its absence (blank) at 37 °C for EC/SG60 3_NFs and EC/SG90 3_NFs. (C) Tensile strength, (D) elongation at break, and (E) Young's modulus values determined from the slope of the common linear region (25–45% strain) of the corresponding stress–strain curves for EC/SG60 3_NFs and EC/SG90 3_NFs, compared with uncoated NAR-NCs_NFs. (F) In vitro release profiles of NAR-NCs_NFs, EC/SG60 3_NFs and EC/SG90 3_NFs in simulated saliva fluid (SSF). Data are presented as mean ± SE (n = 6) for panel B, mean ± SD (n = 4) for panels C–E, and mean ± SD (n = 3) for panel F. Statistical analysis was performed using one-way ANOVA followed by Tukey's multiple comparisons test (*p < 0.05, **p < 0.01, ***p < 0.001, ****p < 0.0001). EC/SG60 3 and EC/SG90 3 correspond to EC/SG volume ratios of 55:45 (v/v) prepared using SG60 and SG90, respectively.

Based on the combined mucoadhesive and mechanical outcomes, EC/SG60 3 and EC/SG90 3 emerged as the most promising prototypes, displaying the highest tensile strength and robust mucoadhesion without compromising flexibility. These two formulations were therefore selected for further integration with the NAR-NCs_NFs platform. A representative image of the coated nanofibres is shown in Fig. 8A.

The mucoadhesive properties of the integrated EC/SG_NFs are presented in Fig. 8B. Both coated systems exhibited a marked increase in detachment force in the presence of mucin compared with the corresponding blank measurements, confirming that the EC/SG coating retained its mucoadhesive properties after integration with the nanofibrous mat. Moreover, EC/SG90 3_NFs displayed a significantly higher detachment force than EC/SG60 3_NFs, indicating stronger interaction with the mucin substrate.

To evaluate the impact of the film coating on the mechanical behaviour of the nanofibrous system, NAR-NCs_NFs were coated with the selected prototypes generating the integrated structures EC/SG_NFs. Representative stress–strain curves are shown in Fig. S3C.

As shown in Fig. 8C–E, coating the nanofibres with either EC/SG60 3 or EC/SG90 3 significantly increased tensile strength, elongation at break and Young's modulus compared with the uncoated NAR-NCs_NFs. These findings indicate that the film layer provides additional structural reinforcement while simultaneously improving the deformability of the integrated platform, without compromising its elastic response.

To assess whether the EC/SG coating affected drug release from the final integrated platform, the *in vitro* release profiles of EC/SG60 3_NFs and EC/SG90 3_NFs were compared with that of the uncoated NAR-NCs_NFs (Fig. 8F). Both coated formulations exhibited release profiles closely resembling that of the uncoated nanofibres, indicating that the EC/SG film did not substantially alter the sustained-release behaviour of the platform. Complete drug release was maintained over approximately 72 h for all formulations, demonstrating that the incorporation of the mucoadhesive film preserved the long-acting release characteristics of the electrospun nanofibres.

The cytocompatibility of the integrated systems EC/SG60 3_NF and EC/SG90 3_NF was evaluated using NHDF, employed as a general human fibroblast model for preliminary biocompatibility screening in accordance with ISO 10993-5 guidelines, as commonly reported in biomaterial studies [43].

**Fig. 9:**
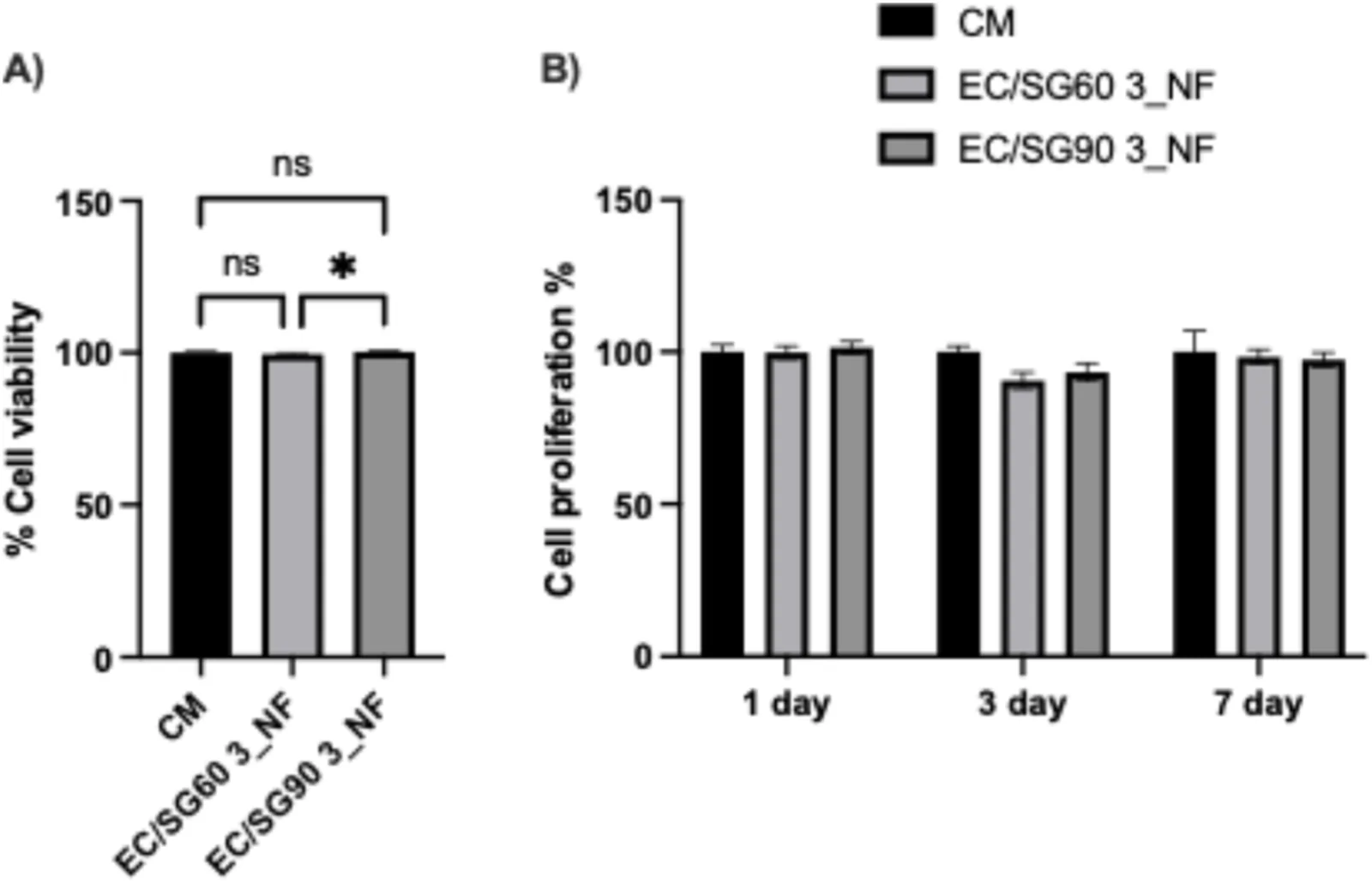
Biological evaluation of the integrated EC/SG_NF systems. (A) Cell viability of normal human dermal fibroblasts (NHDF) following exposure to extracts of EC/SG60 3_NF and EC/SG90 3_NF. (B) NHDF proliferation following direct contact with EC/SG60 3_NF and EC/SG90 3_NF for 1, 3 and 7 days. Complete culture medium (CM) was used as the control. Data are presented as mean ± SD (n = 6). Statistical analysis was performed using one-way ANOVA followed by Tukey's multiple comparisons test (p < 0.05).

Fig. 9A shows the percentage of viable cells after 24 h exposure to the extracts of the two prototypes. Both EC/SG_NF formulations maintained cell viability values close to 100%, with no significant reduction compared to the CM. These results confirm that both integrated systems are non-cytotoxic and compatible with human fibroblasts, supporting their suitability for further development as buccal drug-delivery prototypes.

Long-term proliferation studies further confirmed that continuous exposure to EC/SG_NFs extracts did not negatively affect NHDF growth throughout the 7-day evaluation period. At days 1, 3, and 7, cell proliferation profiles remained comparable to untreated controls, with no statistically significant differences observed between treated and control groups (Fig. 9B). These findings indicate that the integrated systems do not interfere with normal fibroblast growth kinetics under prolonged exposure conditions, further supporting their sustained cytocompatibility. This behaviour is particularly relevant for buccal applications, where prolonged mucosal residence is essential, reinforcing the biological suitability of the developed platform for extended local administration.

## 4 Conclusions

This work presents a multifunctional platform for the local treatment of oral mucositis based on NAR-NCs incorporated into electrospun core–shell NFs and integrated with an EC/SG mucoadhesive film. The proposed strategy addresses key limitations of conventional local therapies by combining enhanced drug dissolution, sustained release, prolonged mucosal residence, and improved handling properties within a single delivery system. NAR-NCs were successfully produced by dual asymmetric centrifugation, yielding stable nanocrystals with preserved crystallinity and enhanced aqueous solubility. Their incorporation into core–shell NFs enabled controlled drug release while preserving the anti-inflammatory activity of NAR. Integration with the EC/SG film further improved the mechanical performance of the system while providing strong mucoadhesion and excellent cytocompatibility, supporting its suitability for buccal application. Overall, the developed platform represents a promising local drug delivery system for oral mucositis. Future studies will include *ex vivo* evaluation using porcine buccal mucosa to investigate mucosal retention, followed by *in vivo* studies to assess local safety and therapeutic efficacy under physiological conditions.

## CRediT authorship contribution statement

**Gaia Zucca:** Writing – original draft, Investigation, Formal analysis, Data curation, Conceptualization. **Barbara Vigani:** Writing – review & editing, Supervision, Methodology, Investigation, Conceptualization, Data curation. **Caterina Valentino:** Writing – review & editing, Methodology, Data curation. **Lucia Lopez-Vidal:** Writing – review & editing, Methodology, Data curation. **Martina Sangalli:** Writing – review & editing, Methodology, Data curation. **Marco Ruggeri:** Writing – review & editing, Data curation. **Giuseppina Sandri:** Resources, Methodology, Data curation. **Silvia Rossi:** Writing – review & editing, Supervision, Resources, Methodology, Data curation. **Alejandro J. Paredes:** Writing – review & editing, Supervision, Resources, Methodology, Conceptualization, Funding acquisition, Project administration.

## Declaration of competing interest

The authors declare that they have no known competing financial interests or personal relationships that could have appeared to influence the work reported in this paper.

## Data Availability

Data will be made available on request.
