# Supplementary material for: Electrospun core–shell nanofibres embedding drug nanocrystals for long-acting mucosal delivery
Source: Mater Today Bio. 2026 Sep 8;40:103653. doi: 10.1016/j.mtbio.2026.103653 (PMC13573742; doi:10.1016/j.mtbio.2026.103653)
Supplement: Supplementary file 1 [file mmc1.docx]

*Supplementary material*

**Electrospun Core–Shell Nanofibres Embedding Drug Nanocrystals for Long-acting Mucosal Delivery**

Gaia Zucca^1^, Barbara Vigani^1^, Caterina Valentino^1^, Lucia Lopez-Vidal^2^, Martina Sangalli^2^, Marco Ruggeri^1^, Giuseppina Sandri^1^, Silvia Rossi^1^, Alejandro J. Paredes^2*^.

*^1^Department of Drug Sciences, University of Pavia, Viale Taramelli 12, 27100 Pavia, Italy*

*^2^School of Pharmacy, Queens University Belfast, Medical Biology Centre, 97 Lisburn Road, Belfast, BT9 7BL, United Kingdom*

***Corresponding author:**

**Alejandro J. Paredes**

Reader in Pharmaceutical Sciences

School of Pharmacy, Queens University Belfast

Medical Biology Centre

97 Lisburn Road, Belfast, BT9 7BL

United Kingdom

E-mail: a.paredes@qub.ac.uk

ORCID: <https://orcid.org/0000-0002-0414-8972>

**Figure S1-** *Schematic illustration of the preparation of naringenin nanocrystals (NAR-NCs) by dual asymmetric centrifugation-assisted wet media milling. Briefly, 100 mg of NAR powder was milled in the presence of 3 mL yttria-stabilised zirconia (YTZP) beads and 7 mL of 1% (w/v) poloxamer 188 (P188) stabiliser solution using a dual asymmetric centrifuge (DAC) at 3000 rpm for 18 min. The resulting nanosuspension was subsequently centrifuged (7379 × g, 10 min) to remove excess free stabiliser and recover the NAR-NCs pellet, which was then resuspended in ultrapure water for further characterisation.*

**Figure S2-** *Schematic illustration of the coaxial electrospinning process used to fabricate core–shell nanofibres (NFs). The core solution consisted of poly(vinyl alcohol)/Sangelose® (PVA/SG, 10/1% w/v), while the shell solution consisted of polycaprolactone (PCL, 30% w/v). Both solutions were delivered through a coaxial spinneret and electrospun under an applied electric field to produce core–shell nanofibres.*

1. **Results**

***Figure S3****- Representative stress–strain curves of the developed formulations. (A) Unloaded core–shell nanofibres (NFs) and naringenin nanocrystal-loaded core–shell nanofibres (NAR-NCs_NFs). (B) Free-standing ethyl cellulose/Sangelose® (EC/SG) films prepared using SG60 or SG90. Formulations EC/SG 1, EC/SG 2 and EC/SG 3 correspond to EC/SG volume ratios of 75:25, 65:35 and 55:45 (% v/v), respectively. (C) NAR-NCs_NFs coated with the selected EC/SG60 3 or EC/SG90 3 films (EC/SG60 3_NFs and EC/SG90 3_NFs, respectively), compared with uncoated NAR-NCs_NFs. One representative stress–strain curve is shown for each formulation.*
